# Cardiometabolic deaths attributable to poor diet among Kuwaiti adults

**DOI:** 10.1371/journal.pone.0279108

**Published:** 2022-12-15

**Authors:** Badreya Al-Lahou, Lynne M. Ausman, José L. Peñalvo, Gordon S. Huggins, Fang Fang Zhang

**Affiliations:** 1 Environment and Life Sciences Research Center, Kuwait Institute for Scientific Research, Kuwait City, Kuwait; 2 Friedman School of Nutrition Science and Policy, Tufts University, Boston, Massachusetts, United States of America; 3 Department of Public Health, Institute of Tropical Medicine, Antwerp, Belgium; 4 Tufts Medical Center and Tufts University School of Medicine, Boston, Massachusetts, United States of America; Texas A&M University College Station, UNITED STATES

## Abstract

**Background:**

Nutrition transition towards a Western diet is happening in parallel with the rapidly increasing rates of cardiovascular disease and its risk factors in Kuwait. The cardiometabolic deaths attributable to poor diet have not been quantified among Kuwaiti adults.

**Methods:**

Using a Comparative Risk Assessment model that incorporated dietary intake data from Kuwait’s first national nutrition survey, number of cardiometabolic deaths from the World Health Organization, and estimated associations of diet with cardiometabolic deaths from the Global Burden of Disease project, we estimated the number and proportion of cardiometabolic deaths attributable to suboptimal intake of 10 dietary factors among Kuwaiti adults ages 25+ years, and by population subgroups.

**Findings:**

An estimated 1,308 (95% uncertainty interval [UI] = 1,228–1,485) cardiometabolic deaths were attributed to suboptimal diet, accounting for 64.7% (95% UI = 60.7%-73.4%) of all cardiometabolic deaths in Kuwait in 2009. The low intake of nuts/seeds was associated with the highest estimated number and proportion of cardiometabolic deaths (n = 380, 18.8%), followed by high intake of sodium (n = 256, 12.6%), low intake of fruits (n = 250, 12.4%), low intake of vegetables (n = 236, 11.7%), low intake of whole grains (n = 201, 9.9%), and high intake of sugar-sweetened beverages (n = 201, 9.9%). The estimated proportions of cardiometabolic deaths attributable to suboptimal diet were higher in men (67.7%) than women (57.8%) and in younger adults aged 25–34 years (84.5%) than older adults aged ≥55 years (55.6%).

**Conclusion:**

Suboptimal dietary intake was associated with a very substantial proportion of cardiometabolic deaths among Kuwaiti adults in 2009, with young adults and men experiencing the largest proportion of diet-associated cardiometabolic deaths in Kuwait.

## Introduction

Heart disease, stroke, and type 2 diabetes, collectively known as cardiometabolic diseases, are the first leading cause of death in Kuwait, accounting for 44% of all deaths in 2016 [1]. The Global Burden of Diseases, Injuries, and Risk Factors (GBD) Study has estimated that a suboptimal diet low in fruits, vegetables, whole grains, nuts, and seeds, and high in processed meats, red meats, and sugar-sweetened beverages (SSBs), contributes to the highest number of deaths from non-communicable diseases (cardiovascular diseases [CVD], type 2 diabetes, and cancers) globally than any other metabolic risk factor [2].

In recent years, dietary intake patterns among Kuwaiti adults are shifting towards Western-style diet due to the influence of urbanization and economic development [3]. High consumption of SSBs, fast foods and other processed foods high in calories, added sugars, sodium, and saturated fats, and low consumption of fruits and vegetables is evident, particularly among the younger generations [3–10] that form the largest proportion of the Kuwaiti population (70% younger than 35 years) [11]. During the same time, obesity, hypertension, and CVD [12–14] have reached an alarming prevalence in Kuwait. Thus, the burden of cardiometabolic diseases is likely to increase in Kuwait as the young population ages [15].

Understanding the impact of the intakes of key dietary factors associated with cardiometabolic deaths in Kuwait will help policymakers and researchers establish priorities for nutrition-related interventions, programs, research, and policies. This study aimed to estimate the number and proportion of CVD and type 2 diabetes deaths attributable to the suboptimal intake of ten dietary factors, individually and combined, overall and by age and sex subgroups in Kuwaiti adults aged 25 years or older.

## Methods

### Study design

We used a population-based comparative risk assessment (CRA) analysis [16] to estimate the cardiometabolic deaths due to suboptimal dietary intake in Kuwait (S1 Appendix). The model incorporated data and corresponding uncertainty on (1) dietary intake among Kuwaiti adults by age and sex from the most recent Kuwait National Nutrition Survey; (2) the optimal intake distribution of dietary factors associated with the lowest risk in epidemiological studies; (3) the etiologic relationships between diet and cardiometabolic deaths from meta-analyses; and (4) disease-specific cardiometabolic mortality data by age and sex from the World Health Organization.

### Current distribution of dietary intake

Current dietary intake distribution was obtained from a nationally representative sample of Kuwaiti adults from the most recent Kuwait National Nutrition Survey (2008–2009) [3]. We incorporated sampling weights that accounted for the complex survey design and survey nonresponse to provide estimates representative of Kuwaiti adults aged 25 years or older. Dietary intake was assessed from an interview-based 24-hour diet recall. Intake of foods and nutrients was adjusted for total energy intake using the density method as the amount per 2000 kcal or percentage of calories (S1 Table).

### Optimal dietary intake distribution

The optimal intake of dietary factors corresponded to the levels associated with the lowest mortality rate in epidemiological studies or the levels observed in low-exposure populations [17]. The standard deviation around each mean was estimated by the GBD study as ± 10% of the mean [18].

### Selection of dietary factors

Based on previous work by the GBD study [16], we included 10 dietary factors in the analysis: fruits, vegetables, whole grains, nuts/seeds, seafood omega-3 fats, polyunsaturated fatty acids (PUFA) as a replacement for saturated fats or carbohydrates, sodium, SSBs, processed meats, and unprocessed red meats (Table 1). Evidence of diet-disease relationships was evaluated by the GBD study using Bradford-Hill criteria [16]. Ten dietary factors have “convincing” or “probable” evidence of a causal relationship with coronary heart disease, strokes, type 2 diabetes, body mass index (BMI), or systolic blood pressure (SBP) from high-quality epidemiological studies [16, 19]. Other dietary factors with insufficient diet-disease causal relationships (e.g., dairy products and calcium) or present overlap with other dietary factors in estimating the joint effect of overall diet (e.g., whole grains and fiber) were not included [16].

**Table 1:** The dietary factors^a^ included in the analysis, their current intake levels among Kuwaiti adults ≥25 years, optimal intake levels, and related cardiometabolic outcomes.

	Current intake^c^		Optimal intake^d^	Cardiometabolic outcome
Dietary factor^b^	Mean (SE)	Median (IQR)	Mean	
**Fruits**_,_ g/d	116.4 (7.8)	27.3 (0–156.5)	300	CHD and stroke
**Vegetables**_,_ g/d	269.5 (11.7)	210.9 (103.3–348.9)	400	CHD and stroke
**Nuts/seeds**, g/d	5.4 (0.75)	0 (0–4.0)	20.2	CHD and diabetes
**Whole grains**, g/d	21.8 (1.7)	0 (0–33.7)	125	CHD, stroke, and diabetes
**Unprocessed red meats**, g/d	37.5 (2.6)	0 (0–50.0)	14.3	Diabetes
**Processed meats**, g/d	3.5 (0.80)	0 (0–0)	0	CHD and diabetes
**Sugar-sweetened beverages**_,_ 8-oz servings/d	0.86 (0.07)	0 (0–1.4)	0	CHD, HHD, stroke, and diabetes
**Polyunsaturated fats**_,_ % energy	4.7 (0.13)	4.1 (2.6–5.9)	11	CHD
**Seafood omega-3 fats**_,_ mg/d	1420 (339)	62.6 (12.6–206)	250	CHD
**Sodium**, mg/d	3282 (72.5)	3000 (2480–3818)	2000	CHD, HHD, other CVD, and stroke

Abbreviations: CHD, coronary heart disease; CVD, cardiovascular disease; HHD, hypertensive heart disease; IQR, interquartile range; SE, standard error.

^a^Dietary factors with presence of “convincing” or “probable” evidence of a causal relationship with cardiometabolic outcomes (CHD, stroke, or diabetes) from high-quality epidemiological studies [16, 17, 19].

^b^Dietary intake: fruits including fresh, frozen, cooked, canned, or dried fruits, excluding fruit juices and pickled or salted fruits. Vegetables including fresh, frozen, cooked, canned, or dried vegetables and legumes, excluding starchy vegetables such as potatoes and corn, vegetable juices, and pickled or salted vegetables. Sugar-sweetened beverages were defined as beverages with ≥50 kcal per 8oz (237g), including carbonated beverages and fruit drinks, excluding 100% juices. Seafood omega-3 fats include intake of Eicosapentaenoic acid and Docosahexaenoic acid.

^c^Current intakes were adjusted for the National Nutrition Survey of the State of Kuwait sampling weights that accounted for complex survey design and nonresponse.

^d^Optimal intakes corresponded to the levels associated with the lowest mortality rate in epidemiological studies or the levels observed in low-exposure populations [17, 18].

### Etiologic relationships between diet and cardiometabolic deaths

This study used age-specific effect sizes (relative risk, RR) of dietary factors on CVD and type 2 diabetes mortality or incidence from published meta-analyses of clinical trials and prospective cohort studies [17, 19]. The effect sizes are similar for both sexes but decline with age [20]. For SSBs, the model incorporated the effect of SSBs on change in BMI and the effect of change in BMI on coronary heart disease, hypertensive heart disease, stroke, and type 2 diabetes deaths [16]. For sodium, the model incorporated the effect of sodium on change in SBP and the effect of change in SBP on coronary heart disease, hypertensive heart disease, other CVD, and stroke deaths [16] (S2 Table).

### National cardiometabolic deaths and BMI and SBP distributions

Disease-specific deaths, by age and sex, in Kuwait were obtained from the World Health Organization 2009 mortality database [21]. The database includes medically-certified deaths reported by Kuwait’s civil registration system; deaths were coded according to the official International Classification of Disease, Tenth Revision (ICD-10). We obtained data on mortality due to (1) heart diseases including: coronary heart disease (I20-I25), hypertensive heart disease (I11), aortic aneurysm (I71), rheumatic heart disease (I01, I020, I05-I09), endocarditis (I33), cardiomyopathy and myocarditis (I40, I42), atrial fibrillation and flutter (I48), peripheral vascular disease (I702, I73), and other CVD and circulatory disease (I00, I1029, I27, I28, I30-I32, I34-I39, I47, I708, I72, I77-I80, I82-I84, I86, except I271, I312, I313); (2) stroke including: Ischemic (I63, I65-I67, I693, G45), hemorrhagic (I60-I62, I690-I692, I674), and other stroke (I64, I694, I698); and, (3) type 2 diabetes (E10-E14, except E102, E112, E122, E132) (S3 Table).

Weight, height, and blood pressure were obtained from the Kuwait National Nutrition Survey (2008–2009) [3, 13]. Weight was measured to the nearest 100 grams by a body composition analyzer (TANITA model TBF 310, Japan) and height was measured to the nearest 1 cm by a vertical stadiometer (Seca 214, Germany) [3]. BMI was calculated as weight in kilograms divided by height in meters squared. Blood pressure was measured using a sphygmomanometer or a “Spengler Electronic Pro M, professional blood pressure monitor” device on the right arm in the sitting position [13].

### Statistical analyses

We adapted the GBD CRA model to estimate the number and proportion of cardiometabolic deaths attributable to suboptimal intake of ten dietary factors, individually and combined [16]. Briefly, the model combined the current and the optimal intake levels of each dietary factor and its disease-specific RR and calculated the population attributable fraction (PAF). The PAF was then multiplied by the number of disease-specific deaths to estimate disease-specific deaths attributable to each dietary factor. The joint PAF of all individual dietary factors was estimated by proportional multiplication of each stratum-specific PAF (S1 Appendix). All analyses were conducted by age (25–34, 35–44, 45–54, 55+ years) and sex (male, female) subgroups. The number of attributable cardiometabolic deaths per 100,000 adults were calculated using the 2009 Kuwaiti adult estimates (S2 Appendix).

The uncertainty of the estimated attributable mortality was quantified using the Monte Carlo simulations [16]. This approach incorporated uncertainties of the current and optimal dietary intake distributions, RRs, prevalence of hypertension (for sodium), and prevalence of overweight (for SSBs) in each age and sex subgroup. The 95% uncertainty intervals (UI) were derived from the 2.5th and 97.5th percentiles of 1000 estimated attributable deaths. All analyses were performed using R software version 3.6.0.

## Results

### Deaths attributed to suboptimal diet

**Fig 1:**
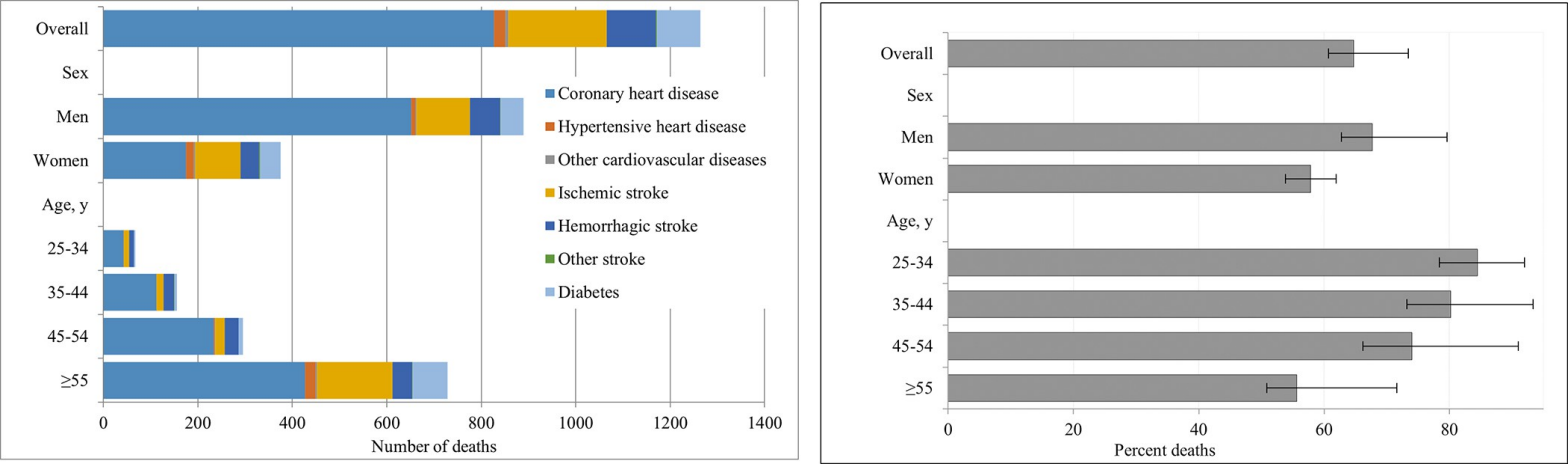
Estimated number (A) and proportion (B) of cardiometabolic deaths attributable to overall suboptimal diet in Kuwait in 2009 by population subgroups. Bars represent the estimated number (A) and percentage (B) of cardiometabolic deaths attributable to overall suboptimal intake of ten dietary factors: low intake of fruits, vegetables, nuts/seeds, whole grains, seafood omega-3 fats, and polyunsaturated fats, and high intake of sodium, unprocessed red meats, processed meats, and sugar-sweetened beverages. Error bars indicate 95% uncertainty intervals.

In 2009, an estimated 1,308 (95% UI = 1228–1485) cardiometabolic deaths were attributable to suboptimal intake of 10 dietary factors including low intake of fruits, vegetables, whole grains, nuts/seeds, seafood omega-3, PUFA, and high intake of unprocessed red meats, processed meats, sodium, and SSBs accounting for 64.7% (95% UI = 60.7–73.4%) of all cardiometabolic deaths in Kuwait (Fig 1 and Table 2). The highest number of cardiometabolic deaths associated with poor diet was for coronary heart disease (n = 827), followed by ischemic stroke (n = 209), hemorrhagic stroke (n = 104), and type 2 diabetes (n = 93) (Table 2). The highest proportion of cardiometabolic deaths associated with poor diet was for hemorrhagic stroke (76.0%), followed by coronary heart disease (70.7%), ischemic stroke (57.7%), and type 2 diabetes (46.2%). The number of attributable disease-specific deaths per 100,000 adults are presented in S4 Table. The highest diet-related cardiometabolic death rate was for coronary heart disease (184 per 100,000 adults), followed by ischemic stroke (46 per 100,000 adults), hemorrhagic stroke (24 per 100,000), and type 2 diabetes (21 per 100,000 adults).

**Table 2:** Estimated number and proportion of cardiometabolic deaths attributable to suboptimal diet among Kuwaiti adults aged ≥25 years in 2009, total and by sex.

	Total		Men		Women	
	Attributable deaths^a^	Population attributable fraction	Attributable deaths^a^	Population attributable fraction	Attributable deaths^a^	Population attributable fraction
Outcome by dietary factor	N (95% UI)	% (95% UI)	N (95% UI)	% (95% UI)	N (95% UI)	% (95% UI)
**Overall diet** ^b^						
CHD	827 (774–876)	70.7 (66.2–74.9)	652 (604–698)	71.5 (66.2–76.6)	175 (158–191)	68.3 (61.5–74.2)
Hypertensive heart disease	25 (19–30)	26.8 (20.8–32.6)	9 (6–11)	24.3 (17.6–31.0)	16 (11–21)	28.4 (19.9–37.1)
Ischemic stroke	209 (185–233)	57.7 (51.0–64.3)	113 (96–129)	57.8 (49.1–65.9)	96 (79–111)	57.5 (47.2–66.3)
Hemorrhagic stroke	104 (96–111)	76.0 (70.1–80.7)	64 (57–69)	77.1 (69.2–83.3)	40 (36–44)	74.3 (65.8–81.4)
Other stroke	2 (2–2)	72.2 (63.3–80.1)	1 (1–1)	85.8 (72.3–93.1)	1 (1–1)	64.0 (50.6–75.0)
Diabetes	93 (80–145)	46.2 (39.8–72.0)	49 (40–96)	48.2 (39.1–95.3)	44 (36–53)	43.8 (36.0–52.7)
*Total CMD*	*1308 (1228–1485)*	*64*.*7 (60*.*7–73*.*4)*	*921 (853–1083)*	*67*.*7 (62*.*7–79*.*6)*	*383 (357–410)*	*57*.*8 (53*.*9–61*.*9)*
**Fruits**, <300 g/d						
CHD	118 (83–155)	10.1 (7.1–13.3)	95 (62–131)	10.4 (6.8–14.4)	23 (13–35)	8.9 (4.9–13.5)
Stroke	132 (113–152)	26.3 (22.4–30.3)	77 (62–92)	27.7 (22.2–33.1)	54 (43–69)	24.4 (19.2–30.8)
*Total CMD*	*250 (209–292)*	*12*.*4 (10*.*3–14*.*4)*	*172 (137–210)*	*12*.*7 (10*.*1–15*.*4)*	*77 (61–97)*	*11*.*7 (9*.*2–14*.*6)*
**Vegetables**, <400 g/d						
CHD	97 (70–128)	8.3 (6.0–11.0)	78 (53–107)	8.5 (5.8–11.7)	19 (10–29)	7.4 (4.0–11.3)
Stroke	138 (103–173)	27.5 (20.6–34.4)	79 (54–105)	28.2 (19.2–37.6)	60 (37–83)	26.7 (16.4–37.2)
*Total CMD*	*236 (192–282)*	*11*.*7 (9*.*5–14*.*0)*	*156 (122–196)*	*11*.*5 (8*.*9–14*.*4)*	*78 (55–104)*	*11*.*8 (8*.*3–15*.*8)*
**Nuts/seeds**, <20.2 g/d						
CHD	354 (293–411)	30.3 (25.1–35.1)	278 (227–334)	30.5 (24.9–36.7)	76 (54–95)	29.5 (21.0–37.0)
Diabetes	26 (19–34)	12.8 (9.3–16.9)	13 (7–19)	12.6 (7.0–18.4)	13 (7–19)	13.2 (7.4–19.2)
*Total CMD*	*380 (321–438)*	*18*.*8 (15*.*9–21*.*7)*	*291 (239–347)*	*21*.*4 (17*.*6–25*.*5)*	*89 (67–109)*	*13*.*5 (10*.*1–16*.*4)*
**Whole grains**, <125 g/d						
CHD	80 (49–112)	6.8 (4.2–9.5)	64 (35–97)	7.0 (3.8–10.6)	15 (7–24)	6.0 (2.6–9.4)
Stroke	81 (60–100)	16.0 (12.0–19.9)	47 (32–61)	17.0 (11.4–21.9)	33 (19–46)	14.9 (8.4–20.7)
Diabetes	41 (31–51)	20.3 (15.2–25.4)	21 (14–28)	20.6 (13.5–28.0)	20 (13–27)	19.8 (12.8–27.2)
*Total CMD*	*201 (163–241)*	*9*.*9 (8*.*0–11*.*9)*	*132 (99–168)*	*9*.*7 (7*.*3–12*.*3)*	*68 (52–86)*	*10*.*3 (7*.*8–13*.*0)*
**Sugar-sweetened beverages**, >0 8-oz servings/d						
CHD	170 (135–209)	14.5 (11.5–17.9)	144 (111–184)	15.8 (12.1–20.2)	25 (18–33)	9.8 (7.0–12.9)
Stroke	6 (5–8)	1.3 (1.0–1.5)	4 (3–5)	1.5 (1.1–1.8)	2 (2–3)	1.0 (0.77–1.3)
Diabetes	24 (16–31)	11.9 (7.9–16.7)	14 (8–22)	13.4 (7.6–21.5)	10 (6–15)	10.0 (6.1–15.0)
*Total CMD*	*201 (165–244)*	*9*.*9 (8*.*2–12*.*1)*	*162 (129–206)*	*11*.*9 (9*.*4–15*.*2)*	*38 (30–48)*	*5*.*8 (4*.*5–7*.*2)*
**Unprocessed red meat**, >14.3 g/d						
Diabetes	14 (7–23)	6.8 (3.2–11.5)	8 (2–15)	7.7 (2.2–15.0)	6 (1–11)	5.8 (1.5–11.4)
*Total CMD*	*14 (7–23)*	*0*.*68 (0*.*32–1*.*1)*	*8 (2–15)*	*0*.*57 (0*.*17–1*.*1)*	*6 (1–11)*	*0*.*88 (0*.*22–1*.*7)*
**Processed meat**, >0 g/d						
CHD	51 (12–522)	4.3 (1.0–44.6)	40 (7–513)	4.4 (0.80–56.2)	9 (1–34)	3.6 (0.46–13.4)
Diabetes	8 (3–99)	4.0 (1.3–49.3)	3 (1–92)	3.2 (0.93–90.9)	4 (1–13)	4.5 (0.66–13.1)
*Total CMD*	*70 (19–561)*	*3*.*5 (0*.*95–27*.*7)*	*55 (11–548)*	*4*.*0 (0*.*80–40*.*3)*	*14 (3–39)*	*2*.*0 (0*.*47–5*.*8)*
**Sodium**, >2000 mg/d						
CHD	143 (109–183)	12.3 (9.3–15.6)	108 (78–143)	11.8 (8.5–15.7)	35 (24–49)	13.5 (9.4–19.2)
Stroke	83 (70–97)	16.6 (14.0–19.3)	43 (34–53)	15.5 (12.2–18.9)	40 (31–51)	17.9 (13.7–22.7)
*Total CMD*	*256 (219–296)*	*12*.*6 (10*.*8–14*.*6)*	*162 (130–200)*	*11*.*9 (9*.*6–14*.*7)*	*93 (77–111)*	*14*.*1 (11*.*7–16*.*8)*
**PUFA replacing carbohydrates**, <11% energy/d						
CHD	161 (125–197)	13.8 (10.7–16.9)	128 (94–162)	14.0 (10.3–17.8)	33 (21–46)	12.9 (8.3–18.0)
*Total CMD*	*161 (125–197)*	*8*.*0 (6*.*2–9*.*8)*	*128 (94–162)*	*9*.*4 (6*.*9–11*.*9)*	*33 (21–46)*	*5*.*0 (3*.*2–7*.*0)*
**Seafood omega-3 fats**, <250 mg/d						
CHD	192 (104–287)	16.4 (8.9–24.6)	146 (63–241)	16.1 (6.9–26.4)	44 (22–69)	17.0 (8.6–26.7)
*Total CMD*	*192 (104–287)*	*9*.*5 (5*.*1–14*.*2)*	*146 (63–241)*	*10*.*8 (4*.*6–17*.*7)*	*44 (22–69)*	*6*.*6 (3*.*3–10*.*4)*

Abbreviations: CHD, coronary heart disease; CMD, cardiometabolic diseases; PUFA, polyunsaturated fatty acids; UI, uncertainty intervals

^a^Attributable deaths were calculated by multiplying the total number of disease-specific deaths in Kuwait in 2009 with the stratum-specific population attributable fraction.

^b^For the overall diet, the population attributable fraction was estimated based on the joint (multiplicative) population attributable fraction for ten dietary factors: fruits, vegetables, whole grains, nuts/seeds, seafood omega-3 fats, PUFA as a replacement of saturated fats or carbohydrates, sodium, sugar-sweetened beverages, processed meat, and unprocessed red meat.

**Fig 2:**
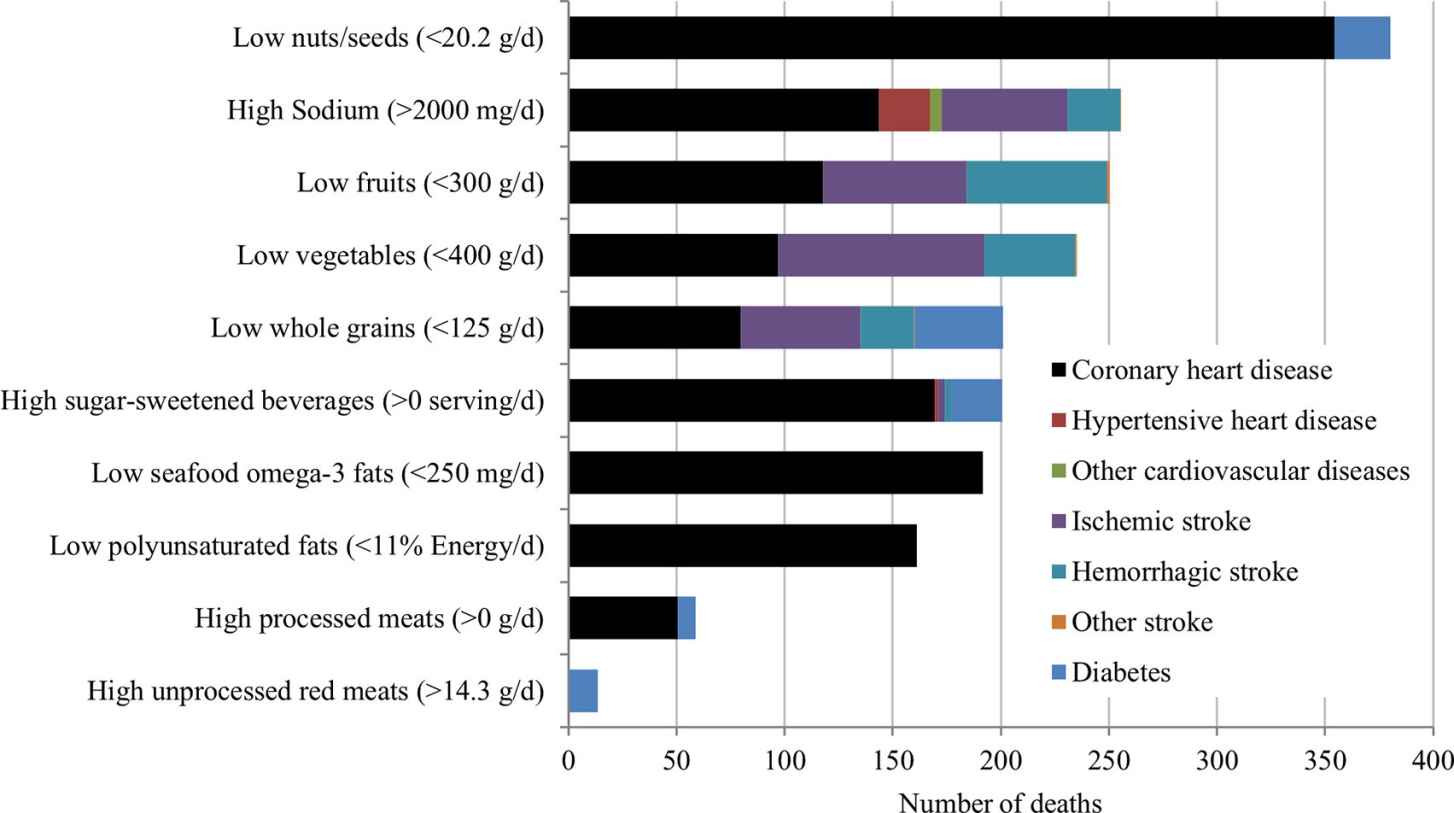
Estimated number of cardiometabolic deaths attributable to suboptimal dietary intake in Kuwait in 2009. Bars represent the estimated number of cardiometabolic deaths attributable to intake of individual dietary factors compared to the optimal intake (numbers in parentheses).

Among individual dietary factors, low intake of nuts/seeds was the largest contributor to cardiometabolic deaths (n = 380 estimated deaths, 18.8% of all cardiometabolic deaths) (Fig 2 and Table 2). The second largest individual dietary contributor was high intake of sodium (n = 256, 12.6%) followed by low intake of fruits (n = 250, 12.4%) and vegetables (n = 236, 11.7%). High intake of SSBs and low intake of whole grains were each associated with 201 estimated deaths accounting for 9.9% of all cardiometabolic deaths. The lowest estimated proportions of deaths due to an individual dietary factor were for high intake of unprocessed red meats and processed meats, 0.68% (n = 14) and 3.5% (n = 70), respectively.

### Attributable deaths by age and sex

By sex, the estimated deaths associated with overall suboptimal dietary intake was higher in men than women (Fig 1 and Table 2). The five dietary factors associated with the highest cardiometabolic deaths in men were low intake of nuts/seeds (n = 291 estimated cardiometabolic deaths, 21.4% of all cardiometabolic deaths), low intake of fruits (n = 172, 12.7%), high intake of sodium (n = 162, 11.9%), high intake of SSBs (n = 162, 11.9%), and low intake of vegetables (n = 156, 11.5%). In women, high intake of sodium (n = 93, 14.1%) and low intake of nuts/seeds (n = 89, 13.5%), vegetables (n = 78, 11.8%), fruits (n = 77, 11.7%), and whole grains (n = 68, 10.3%), were the five dietary factors associated with the largest number of cardiometabolic deaths.

The number of cardiometabolic deaths attributable to overall suboptimal diet was higher in older adults (aged 55 years or older) compared to younger adults (25–34 years), whereas the proportion of cardiometabolic deaths was higher in younger adults compared to older adults at 84.5% vs. 55.6%, respectively (Fig 1 and Table 3). The contribution of individual dietary factors on cardiometabolic deaths was different by age group. Low intake of nuts/seeds was the largest contributor to cardiometabolic deaths for all ages except younger adults (25–34 years) for whom high intake of SSBs was the leading dietary factor. Low fruit intake, high sodium intake, and low seafood omega-3 fats were the second leading dietary risk factors in adults aged 25–44, 45–54, and 55 years or older, respectively. The third leading dietary risk factor was low nuts/seeds intake in adults aged 25–34, high SSBs intake in adults 35–44 years, low fruit intake in adults 45–54 years, and low vegetable intake in adults 55 years or older.

**Table 3:** Estimated number and proportion of cardiometabolic deaths attributable to dietary factors among Kuwaiti adults aged ≥25 years in 2009 by age group.

	25–34 y		35–44 y		45–54 y		55+ y	
	Attributable deaths^a^	Population attributable fraction	Attributable deaths^a^	Population attributable fraction	Attributable deaths^a^	Population attributable fraction	Attributable deaths^a^	Population attributable fraction
Outcome by dietary factor	N (95% UI)	% (95% UI)	N (95% UI)	% (95% UI)	N (95% UI)	% (95% UI)	N (95% UI)	% (95% UI)
**Overall diet** ^b^								
CHD	42 (39–44)	87.6 (81.6–92.3)	112 (99–123)	81.1 (72.0–88.8)	233 (206–256)	77.0 (68.0–84.5)	427 (385–474)	62.9 (56.7–69.7)
Hypertensive heart disease	1 (0–1)	28.6 (10.4–46.7)	0 (0–0)	32.7 (17.3–47.7)	2 (2–3)	30.2 (21.7–37.8)	21 (16–27)	26.4 (19.8–33.0)
Ischemic stroke	11 (10–12)	79.8 (69.3–86.9)	15 (13–16)	74.9 (65.9–81.8)	21 (17–23)	66.6 (55.8–74.9)	160 (136–184)	54.0 (45.7–62.0)
Hemorrhagic stroke	11 (10–11)	89.2 (79.3–94.1)	23 (20–24)	86.6 (75.5–92.9)	29 (25–32)	78.9 (67.6–86.7)	41 (34–46)	65.5 (55.4–74.2)
Other stroke	0	0	1 (1–1)	1 (1–1)	0	0	1 (1–1)	64.0 (50.6–75.0)
Diabetes	3 (2–3)	91.5 (74.6–1.0)	5 (4–6)	84.6 (58.4–98.2)	9 (8–13)	54.9 (46.2–79.2)	74 (61–126)	42.3 (34.8–72.3)
*Total CMD*	*73 (67–79)*	*84*.*5 (78*.*4–92*.*0)*	*158 (144–184)*	*80*.*2 (73*.*2–93*.*4)*	*298 (266–366)*	*74*.*0 (66*.*2–91*.*0)*	*744 (680–957)*	*55*.*6 (50*.*9–71*.*6)*
**Fruits**, <300 g/d								
CHD	9 (5–13)	18.0 (9.7–26.6)	23 (10–36)	16.7 (7.4–25.9)	34 (15–57)	11.3 (4.9–18.7)	51 (26–76)	7.5 (3.9–11.2)
Stroke	13 (10–15)	49.8 (39.5–57.8)	24 (18–28)	50.5 (37.4–59.8)	26 (18–32)	38.1 (26.8–47.6)	70 (53–89)	19.4 (14.7–24.5)
*Total CMD*	*22 (17–26)*	*25*.*0 (19*.*3–30*.*6)*	*47 (33–60)*	*23*.*8 (16*.*8–30*.*3)*	*60 (40–83)*	*15*.*0 (9*.*9–20*.*8)*	*120 (90–150)*	*9*.*0 (6*.*7–11*.*2)*
**Vegetables**, <400 g/d								
CHD	7 (4–11)	15.0 (7.7–22.3)	17 (8–26)	12.2 (5.8–19.0)	28 (14–43)	9.3 (4.6–14.3)	45 (24–70)	6.6 (3.6–10.4)
Stroke	12 (8–15)	45.1 (30.4–58.1)	18 (11–24)	38.4 (24.2–50.8)	21 (13–29)	30.9 (18.7–42.1)	88 (53–120)	24.4 (14.6–33.1)
*Total CMD*	*19 (14–24)*	*21*.*9 (16*.*3–27*.*7)*	*35 (24–46)*	*17*.*7 (12*.*0–23*.*2)*	*49 (32–66)*	*12*.*2 (8*.*1–16*.*3)*	*134 (95–173)*	*10*.*0 (7*.*1–12*.*9)*
**Nuts/seeds**, <20.2 g/d								
CHD	19 (14–24)	39.1 (28.9–49.8)	49 (32–65)	35.6 (23.2–46.8)	101 (68–131)	33.4 (22.3–43.4)	186 (138–231)	27.3 (20.4–34.0)
Diabetes	1 (0–1)	18.5 (11.3–25.5)	1 (0–1)	15.4 (6.9–23.5)	3 (2–4)	15.2 (9.6–20.6)	22 (15–30)	12.4 (8.3–17.0)
*Total CMD*	*19 (14–24)*	*22*.*5 (16*.*7–28*.*4)*	*50 (33–66)*	*25*.*5 (16*.*6–33*.*3)*	*104 (70–134)*	*25*.*8 (17*.*5–33*.*3)*	*208 (161–254)*	*15*.*5 (12*.*0–19*.*0)*
**Whole grains**, <125 g/d								
CHD	5 (2–9)	11.2 (4.1–18.4)	13 (4–22)	9.7 (2.9–16.2)	24 (6–42)	7.9 (2.0–13.9)	37 (14–60)	5.5 (2.1–8.8)
Stroke	7 (4–9)	26.4 (17.0–33.7)	11 (7–14)	23.4 (14.9–30.5)	13 (9–18)	19.8 (12.7–26.2)	49 (29–67)	13.6 (8.1–18.6)
Diabetes	1 (1–1)	35.4 (25.3–44.1)	2 (1–2)	32.0 (22.9–40.5)	4 (3–6)	26.5 (18.4–33.7)	33 (23–43)	19.0 (13.1–24.8)
*Total CMD*	*13 (9–17)*	*15*.*4 (10*.*6–19*.*8)*	*26 (16–36)*	*13*.*4 (8*.*3–18*.*3)*	*42 (23–61)*	*10*.*4 (5*.*8–15*.*1)*	*119 (88–151)*	*8*.*9 (6*.*6–11*.*3)*
**Sugar-sweetened beverages**, >0 8-oz servings/d								
CHD	19 (13–26)	40.0 (27.6–53.7)	35 (21–52)	25.4 (15.6–37.4)	53 (32–80)	17.5 (10.6–26.3)	61 (39–87)	8.9 (5.7–12.8)
Stroke	1 (1–2)	5.2 (3.8–6.8)	1 (1–2)	2.9 (1.9–3.9)	1 (1–2)	1.7 (1.2–2.5)	2 (2–3)	0.67 (0.46–0.94)
Diabetes	1 (0–2)	47.0 (28.6–67.4)	2 (1–3)	29.8 (17.4–45.8)	3 (2–5)	18.6 (11.3–27.5)	17 (10–28)	9.9 (5.5–15.7)
*Total CMD*	*22 (16–29)*	*25*.*8 (18*.*5–33*.*6)*	*38 (25–55)*	*19*.*3 (12*.*6–27*.*7)*	*58 (36–84)*	*14*.*4 (9*.*0–20*.*9)*	*82 (58–110)*	*6*.*1 (4*.*3–8*.*2)*
**Unprocessed red meats**, >14.3 g/d								
Diabetes	0 (0–1)	12.9 (4.0–26.0)	0 (0–1)	6.9 (2.8–12.8)	1 (0–2)	5.3 (2.1–10.0)	12 (5–22)	6.9 (2.8–12.3)
*Total CMD*	*0 (0–1)*	*0*.*45 (0*.*14–0*.*91)*	*0 (0–1)*	*0*.*21 (0*.*08–0*.*39)*	*1 (0–2)*	*0*.*22 (0*.*09–0*.*42)*	*12 (5–22)*	*0*.*90 (0*.*36–1*.*6)*
**Processed meats**, >0 g/d								
CHD	12 (1–48)	25.3 (2.1–99.6)	13 (2–127)	9.5 (1.4–91.8)	2 (0–277)	0.58 (0.14–91.5)	4 (0–470)	0.55 (0–69.1)
Diabetes	2 (0–3)	66.7 (8.4–100)	4 (0–6)	60.4 (3.0–95.4)	1 (0–9)	4 (1–53)	1 (0–89)	0.72 (0–50.9)
*Total CMD*	*14 (3–49)*	*16*.*3 (3*.*2–57*.*1)*	*16 (4–131)*	*8*.*2 (2*.*2–66*.*4)*	*3 (1–278)*	*0*.*69 (0*.*25–69*.*2)*	*7 (1–472)*	*0*.*53 (0*.*06–35*.*3)*
**Sodium**, >2000 mg/d								
CHD	5 (2–10)	10.8 (3.9–21.1)	16 (7–31)	11.6 (4.8–22.3)	42 (23–67)	13.7 (7.5–22.2)	79 (57–108)	11.6 (8.3–15.8)
Stroke	4 (3–6)	15.8 (10.1–21.6)	8 (6–12)	17.8 (12.2–25.0)	13 (10–17)	19.8 (14.7–25.3)	57 (45–71)	15.8 (12.4–19.6)
*Total CMD*	*10 (7–16)*	*11*.*9 (7*.*7–18*.*2)*	*25 (15–40)*	*12*.*8 (7*.*7–20*.*4)*	*58 (39–84)*	*14*.*4 (9*.*6–20*.*9)*	*161 (134–193)*	*12*.*0 (10*.*0–14*.*4)*
**Polyunsaturated fats replacing carbohydrates**, <11% energy/d								
CHD	9 (5–12)	18.3 (11.1–24.8)	24 (13–33)	17.1 (9.1–23.6)	47 (27–67)	15.5 (8.9–22.0)	82 (52–111)	12.1 (7.6–16.4)
*Total CMD*	*9 (5–12)*	*10*.*2 (6*.*2–13*.*9)*	*24 (13–33)*	*12*.*0 (6*.*4–16*.*5)*	*47 (27–67)*	*11*.*7 (6*.*7–16*.*6)*	*82 (52–111)*	*6*.*1 (3*.*9–8*.*3)*
**Seafood omega-3 fats**, <250 mg/d								
CHD	11 (3–21)	22.9 (7.1–43.9)	24 (3–64)	17.6 (1.9–46.2)	68 (8–131)	22.5 (2.6–43.1)	81 (36–164)	12.0 (5.4–24.1)
*Total CMD*	*11 (3–21)*	*12*.*8 (4*.*0–24*.*5)*	*24 (3–64)*	*12*.*3 (1*.*3–32*.*4)*	*68 (8–131)*	*17*.*0 (1*.*9–32*.*5)*	*81 (36–164)*	*6*.*1 (2*.*7–12*.*2)*

Abbreviations: CHD, coronary heart disease; CMD, cardiometabolic diseases; PUFA, polyunsaturated fatty acids; UI, uncertainty intervals

^a^Attributable deaths were calculated by multiplying the total number of disease-specific deaths in Kuwait in 2009 with the stratum-specific population attributable fraction.

^b^For the overall diet, the population attributable fraction was estimated based on the joint (multiplicative) population attributable fraction for ten dietary factors: fruits, vegetables, whole grains, nuts/seeds, seafood omega-3 fats, PUFA as a replacement of saturated fats or carbohydrates, sodium, sugar-sweetened beverages, processed meat, and unprocessed red meat.

## Discussion

Using the CRA approach, we estimated that more than 1,300 cardiometabolic deaths in Kuwait in 2009 were attributable to suboptimal intake of ten dietary factors and accounted for 64.7% of all cardiometabolic deaths among Kuwaiti adults. The dietary factors associated with the largest cardiometabolic burden were low intake of nuts/seeds, fruits, and vegetables, and high intake of sodium. Men and younger adults had higher proportions of diet-related cardiometabolic deaths compared to women and older adults. These findings highlight the potential priorities for reducing cardiometabolic burden in Kuwait.

The estimated proportion of cardiometabolic deaths attributable to suboptimal diet in our study (64.7%) was higher than what was estimated for cardiometabolic deaths attributable to other cardiometabolic risk factors including high systolic blood pressure (>115 mmHg, 47.8%), overweight/obesity (BMI >23 kg/m^2^, 36.2%), high fasting plasma glucose (>5.3 mmol/L, 28.5%), and high serum total cholesterol (>4 mmol/L, 21.3%) [22]. As a suboptimal diet is the leading risk factor for cardiometabolic deaths in Kuwait, urgent measures including policies, programs, and interventions are needed to improve the unhealthy dietary intake of this population.

Overall, the low intake of nuts/seeds, fruits, and vegetables and the high intake of sodium were the dietary factors estimated to be associated with the highest number of cardiometabolic deaths among Kuwaiti adults. The contribution of individual dietary factors to cardiometabolic deaths differed by population subgroups. Low intake of nuts/seeds was the dietary factor estimated to be associated with the highest number of cardiometabolic deaths among men and adults aged 35 years or older. High intake of sodium and high intake of SSBs were estimated to be associated with the largest cardiometabolic burden in women and in young adults aged 25–34 years, respectively; these are likely due to the abundance of fast food outlets in Kuwait [23] and their high sodium and sugar contents. Fast foods and SSBs consumption are prevalent in the younger generation in Kuwait, including among adolescents (14–19 years) [4], high school [24] and college students [9, 23], and younger adults [10]. Our findings highlight the urgent need to target these dietary factors and integrate age and sex differences, to achieve greater benefits in reducing cardiometabolic burden in the Kuwaiti population.

In Kuwait, overweight/obesity prevalence has increased from 75% in 2006 among Kuwaiti adults aged 18–65 years to 77% in 2014 among Kuwaiti adults aged 20–69 years [12]. Diabetes prevalence (fasting plasma glucose ≥7 mmol/L or a current use of glucose-lowering medications) among Kuwaiti adults has also increased from 12.4% in 2006 to 16.7% in 2014 [14]. Many factors can explain the increase in prevalence of CVD risk factors including genetic susceptibility, sedentary lifestyle, and poor dietary intake [25]. Researchers have observed a nutrition transition in Kuwait [3] that may help explain the prevalence of metabolic risk factors that place Kuwait among the countries with the highest burden of obesity and diabetes in the world [12, 14]. Few prior studies in Kuwait have examined specific aspects of Kuwaiti’s diet, such as specific foods or nutrients. These studies have shown a high consumption of unhealthy dietary factors such as: fast foods, SSBs, sweets, potato chips, and sodium, combined with a low consumption of fruits and vegetables, across all age groups in Kuwait [3, 4, 6, 8–10, 23, 26]. This is believed to be due to the economic development and urbanization in Kuwait that have increased the availability of westernized and fast foods. This has played a substantial role in creating unhealthy environments and changing the dietary habits of the population [3, 7]. Lack of nutritional knowledge has also contributed to the increase in harmful dietary patterns [27–29]. Policies are needed to create healthier food environments in Kuwait and to increase nutritional awareness, which could help improve dietary patterns among the population.

The proportion of cardiometabolic deaths attributable to the suboptimal intake of dietary factors estimated in this study (64.7%) was higher than what was reported in a previous study of dietary factors in Kuwaiti adults (57.1%) [22]. Our findings build on that earlier work [22], using several approaches. Our study incorporated updated optimal levels of dietary factors intake, updated effect sizes on the association between diet and disease, and BMI-mediated effects of SSBs intake and SBP-mediated effects of sodium intake on cardiometabolic deaths. Our study also used individual-level dietary data to find the consumption distribution of all dietary factors, rather than using a combination of availability data (food balance sheet) and individual-level data, because the amount of food available to consume is higher than the actual intake [22]. Both studies’ estimates for Kuwait were higher than the estimated 45.4% of all cardiometabolic deaths attributed to suboptimal diet in 2012 among US adults aged 25 years or older [16], indicating the potential for greater targeting of dietary factors in Kuwait.

Consistent with studies in the US [16], Brazil [30], and Korea [31], we found that men and younger adults had higher proportions of cardiometabolic deaths attributable to overall suboptimal dietary intake compared to women and older adults. The observed difference in diet-related cardiometabolic burden can be explained by the unhealthier dietary intake of these subgroups. The diet-disease effect sizes used in the analysis also, in part, explain the observed age difference in mortality, as there is a declining effect of poor dietary intake on mortality with age [17, 19]. The age and sex disparities observed in our study could guide policymakers to target these at-risk groups, especially the younger generation. Kuwait is a country with approximately 70% of its population younger than 35 years of age [11]. The younger population of Kuwaitis will likely have an increasing diet-related cardiometabolic burden as they age [15] unless the burden is addressed through effective measures to improve the dietary intake of the population.

The current study has several limitations that should be considered. First, the current dietary intake was estimated based on a single 24-hour dietary recall; although it does not provide usual intake at the individual level, a single dietary recall does provide unbiased estimates of population subgroups. The dietary recall was interview-administered, which minimizes measurement errors, and dietary intake was adjusted for energy intake to further reduce any potential measurement errors. Second, due to the small sample size, we combined adults aged 55 years and older in one group, and this may result in overestimation of cardiometabolic burden. However, the Kuwaiti population is young with only 6.9% of the total population aged 55 years or older (2.3% aged 55–59 years, 1.7% aged 60–64 years, and 2.9% aged ≥65 years). Third, the relative risks used are from meta-analyses of clinical trials and prospective observational studies adjusted for major confounders; however, residual confounding cannot be excluded. Fourth, the estimation of the overall poor dietary intake on mortality may be overestimated due to the use of the joint PAF that assumes independence of the intake of individual dietary factors; however, a GBD study using a similar approach in estimating the joint PAF has shown that the overestimation is likely to be small [16]. Finally, the cardiometabolic burden attributable to diet in this study was estimated based on dietary intake data from ten years ago. The current dietary consumption pattern among Kuwaiti adults may have changed, and this would affect current estimates. Despite these limitations, the lack of recent individual-level dietary data for the Kuwaiti population means the findings of this study should be a key source of information for the government. The lack of research on dietary patterns also suggests that the government should conduct more diet-related studies to evaluate and track diet, formulate policies and plans, measure the progress of programs and guidelines, and monitor diet-disease burden.

## Conclusion

In 2009, an estimated 1,308 cardiometabolic deaths among Kuwaiti adults were attributable to suboptimal intake of ten dietary factors, accounting for 64.7% of all cardiometabolic deaths in Kuwait. Low intake of nuts/seeds, high intake of sodium, and low intake of fruits and vegetables were associated with the highest cardiometabolic burden. Men and young adults had the largest estimated proportions of diet-associated cardiometabolic deaths. Our findings can help policymakers set priorities, plan programs, and develop policies and recommendations to improve the diet of the Kuwaiti population. Implementing these actions will help shift the Kuwaiti diet towards the optimal intake levels, therefore reducing cardiometabolic burden.

## Supporting information

S1 File (PDF)

S1 Appendix Comparative risk assessment model. (PDF)

S2 Appendix Attributable cardiometabolic deaths Per 100,000 adults. (PDF)

S1 Table Consumption levels of the dietary factors included in the analysis among Kuwaiti adults ≥25 years in 2009, by age and sex. (PDF)

S2 Table Relative Risks (RR) of the relationship between dietary factors and cardiometabolic outcomes. (PDF)

S3 Table Cardiometabolic deaths in Kuwait in 2009, total and by age and sex. (PDF)

S4 Table Estimated cardiometabolic deaths (per 100,000 adults) attributable to suboptimal diet among Kuwaiti adults ≥25 years in 2009, total and by age and sex. (PDF)
