# Supplementary material for: Cardiometabolic deaths attributable to poor diet among Kuwaiti adults
Source: PLoS One. 2022 Dec 15;17(12):e0279108. doi: 10.1371/journal.pone.0279108 (PMC9754186; doi:10.1371/journal.pone.0279108)
Supplement: S1 File — (PDF) [file pone.0279108.s001.pdf]

Supporting information references

1. Micha R, Penalvo JL, Cudhea F, Imamura F, Rehm CD, Mozaffarian D. Association Between Dietary Factors and Mortality From Heart Disease, Stroke, and Type 2 Diabetes in the United States. JAMA. 2017;317(9):912-24.

2. Zaghloul S, Al-Hooti SN, Al-Hamad N, Al-Zenki S, Alomirah H, Alayan I, et al. Evidence for nutrition transition in Kuwait: over-consumption of macronutrients and obesity. Public Health Nutr. 2013;16(4):596-607.

3. Lim SS, Vos T, Flaxman AD, Danaei G, Shibuya K, Adair-Rohani H, et al. A comparative risk assessment of burden of disease and injury attributable to 67 risk factors and risk factor clusters in 21 regions, 1990-2010: a systematic analysis for the Global Burden of Disease Study 2010. Lancet. 2012;380(9859):2224-60.

4. Central Statistical Bureau. Annual Statistical Abstract 2015-206. Chapter 3: Population 2019 [Available from: https://www.csb.gov.kw/Pages/Statistics_en?ID=18&ParentCatID=2.

5. Micha R, Kalantarian S, Wirojratana P, Byers T, Danaei G, Elmadfa I, et al. Estimating the global and regional burden of suboptimal nutrition on chronic disease: methods and inputs to the analysis. Eur J Clin Nutr. 2012;66(1):119-29.

6. Shulkin M, Micha R, Rao M, Singh G, Mozaffarian D. Major dietary risk factors for cardiometabolic disease: current evidence for causal effects and effect sizes from the Global Burden of Diseases (GBD) 2015 study. Circulation. 2016;133:AP279.

7. World Health Organization. WHO Mortality Database 2019 [Available from: https://www.who.int/healthinfo/mortality_data/en/.
