# Supplementary material for: Cardiometabolic deaths attributable to poor diet among Kuwaiti adults
Source: PLoS One. 2022 Dec 15;17(12):e0279108. doi: 10.1371/journal.pone.0279108 (PMC9754186; doi:10.1371/journal.pone.0279108)
Supplement: S1 Appendix — (PDF) [file pone.0279108.s002.pdf]

S1 Appendix. Comparative Risk Assessment Model

A comparative risk assessment model [1] of the Global Burden of Disease Study was used to estimate the number and proportion of cardiovascular disease and type 2 diabetes deaths attributable to the intake of ten dietary factors, individually and combined, by age (25-34, 35-44, 45-54, 55+ years) and sex (male, female) subgroups. Using this model, the population attributable fraction (PAF) was estimated for each dietary factor and disease-specific outcome by age and sex subgroups using three main inputs: the dietary factor (P) and its lowest (n) and highest (m) level of intake (x); the relative risk (RR) of dietary factor on each disease-specific outcome; and, the optimal dietary intake distribution (P′).

$$PAF_{Age,Sex} = \frac{\int_n^m RR(x)P(x) - \int_n^m RR(x)P'(x)dx}{\int_n^m RR(x)P(x)dx}$$

P(X)

The distribution of dietary factors was estimated from a single interview-administered 24-hour dietary recall of Kuwaiti adults aged 25 years or older who participated in the Kuwait National Nutrition Survey. We did not include pregnant and lactating women (n=32) and adults with missing (n=2) or implausible dietary data (n=7, defined as mean energy intake above or below three standard deviations) in estimating national intake levels. A final sample of 886 participants was used to estimate foods and nutrients intake by age (25-34, 35-44, 45-54, 55+ years) and sex (male, female) subgroups. Survey weights accounted for the complex survey design and survey nonresponse were incorporated in the analyses to provide estimates representative of Kuwaiti adults aged 25 years or older [2]. Because the distributions of most foods and nutrients were skewed, especially for nuts/seeds and processed meats, they were modeled using the gamma distribution [1].

RR(X)

RR(X) is defined to be

$$\begin{cases} \exp\left(\beta\left(x - y(x)\right)\right) & : x - y(x) \geq 0 \\ 1 & : x - y < 0 \end{cases}$$

Where (β) is the change in log RR per unit of intake, (x) is the current intake level, and (y) is the optimal intake level. The RR increases exponentially as distance from the optimal intake level increases, that there is no risk associated with intake beyond the optimal intake level.

Joint PAF

Summing the individual PAFs would result in overestimation of the joint PAF;[3] therefore, for each stratum and outcome, joint PAF of the effect of overall dietary factors (suboptimal diet) was estimated by proportional multiplication, using the following formula:

$$PAF_{overall} = 1 - \prod_{P=1}^{R}(1 - PAF_P)$$

Where P is each dietary factor and R is the number of dietary factors (=ten). Since dietary factors may be partly correlated, validity analyses were conducted by Micha et al. to compare the effect sizes of overall dietary patterns to each of their individual dietary components [1]. Findings show that the effect sizes of dietary patterns and their individual dietary components are very similar, and bias if present is less likely to be large [1].

Monte Carlo Simulations

The uncertainty of the estimated PAFs was quantified using the Monte Carlo simulations [1]. This approach incorporates stratum specific uncertainty of the current and optimal dietary intake distributions and RRs estimates. For sodium, it additionally incorporates the uncertainty in the prevalence of hypertension, and for sugar-sweetened beverages, the uncertainty in the prevalence

of overweight. For each diet-outcome and stratum, this approach draws randomly 1000 times from the normal distribution of (1) estimate of disease-specific log(RR), (2) estimate of intake mean, and (3) estimate of the prevalence of hypertension. Draws less than zero are changed to zero (for proportions) or 0.00001 (for mean intake), and draws of proportions greater than one are changes to one. Each set of random draws is used to calculate the PAFs and associated deaths.
