# Supplementary material for: Cardiometabolic deaths attributable to poor diet among Kuwaiti adults
Source: PLoS One. 2022 Dec 15;17(12):e0279108. doi: 10.1371/journal.pone.0279108 (PMC9754186; doi:10.1371/journal.pone.0279108)
Supplement: S2 Appendix — (PDF) [file pone.0279108.s003.pdf]

S2 Appendix. Attributable Cardiometabolic Deaths Per 100,000 Adults

To calculate the number of cardiometabolic deaths per 100,000 adults, the number of Kuwaiti adults in 2009 was used to match the data used in the comparative risk assessment model.

First: Estimating Number of Adults in 2009

The census data available in the Kuwait Central Statistical Bureau [4] were for the years 2005 and 2011. Therefore, we estimated the number of adults in 2009 by first calculating the population change from 2005 to 2011. Then, we divided the number by six to get the population change per year. Then, we used the change per year and the 2005 census data to estimate the adults in 2009.

For example:

Change in total adults 25+ y from 2005 to 2011= ([2011 census data – 2005 census data] / 2005 census data) = 0.36

Change in total adults 25+ y per year= 0.36 / 6 = 0.0600677

Estimated number of adults 25+ y in 2009= 2005 census data * change per year * 4 years [2005-2009]) + 2005 census data = 450,427

	**2005 census data**[a]	**2011 census data**[a]	**Population change from 2005 to 2011**	**Population change per year**	**Estimated population in 2009**[b]
**Total adults**	363,168	494,056	0.3604062	0.0600677	450,427
**Sex**					
Men	158,639	220,022	0.3869351	0.0644892	199,561
Women	204,529	274,034	0.3398296	0.0566383	250,866
**Age group**, year					
25-34	122,081	158,818	0.3009232	0.0501539	146,572
35-44	96,489	125,572	0.3014126	0.0502354	115,878
45-54	62,610	90,449	0.4446414	0.0741069	81,169
55+	59,332	86,167	0.4522854	0.0753809	77,222

[a]The census data were obtained from the Kuwait Central Statistical Bureau [4].
[b]The adult population in 2009 was estimated from the 2005 and 2011 census data.

Second: Calculating Death Rates per 100,000 Adults

To calculate disease-specific deaths per 100,000 adults in Kuwait, we divided the number of disease-specific deaths in a specific group by the number of adults in that specific group then we multiplied the number by 100,000. All calculations were repeated to provide age- and sex-specific cardiometabolic death rates.
