# Supplementary material for: Cardiometabolic deaths attributable to poor diet among Kuwaiti adults
Source: PLoS One. 2022 Dec 15;17(12):e0279108. doi: 10.1371/journal.pone.0279108 (PMC9754186; doi:10.1371/journal.pone.0279108)
Supplement: S1 Table — (PDF) [file pone.0279108.s004.pdf]

S1 Table. Consumption levels[a] of the dietary factors included in the analysis among Kuwaiti adults ≥25 years in 2009, by age and sex

	Sex		Age group			
**Dietary factor**	**Men**	**Women**	**25-34 y**	**35-44 y**	**45-54 y**	**55+ y**
**Fruits**[b], g/d	112.0 (10.1)	120.2 (11.8)	74.2 (12.5)	112.6 (18.9)	143.5 (17.4)	212.9 (17.6)
**Vegetables**[c], g/d	228.7 (12.6)	303.5 (16.0)	232.4 (23.5)	277.9 (19.3)	293.0 (17.4)	339.3 (28.9)
**Nuts/seeds**, g/d	4.8 (0.66)	5.9 (1.1)	5.2 (0.83)	7.8 (3.1)	6.3 (0.98)	4.1 (0.78)
**Whole grains**, g/d	17.9 (1.8)	25.0 (2.5)	11.6 (2.1)	19.8 (2.7)	28.9 (5.0)	37.8 (3.7)
**Sugar-sweetened beverages**[d], 8-oz serving/d	0.91 (0.11)	0.82 (0.07)	1.2 (0.11)	0.73 (0.10)	0.50 (0.07)	0.35 (0.07)
**Unprocessed red meats**[e], g/d	49.9 (4.8)	27.1 (2.8)	46.8 (7.2)	33.3 (6.1)	25.1 (4.3)	44.4 (6.5)
**Processed meats**[f], g/d	4.0 (1.4)	3.1 (0.85)	4.4 (1.5)	2.9 (1.2)	2.1 (0.99)	0.26 (0.19)
**Sodium**, mg/d	3148 (64.5)	3394 (113.3)	3230 (115)	3224 (169)	3460 (133)	3238 (77.6)
**Polyunsaturated fats**, % energy	4.5 (0.17)	4.9 (0.20)	4.7 (0.23)	4.8 (0.25)	5.0 (0.27)	4.3 (0.39)
**Seafood omega-3 fats**[g], mg/d	1243 (289.3)	1567 (448.4)	1448 (425)	862 (317)	1813 (775)	1226 (292)

[a]Values are weight-adjusted means and SEs and were adjusted for energy using the density method as an amount per 2000 kcal or as a percentage of energy.
[b]Fruits including fresh, frozen, cooked, canned, or dried fruits, excluding fruit juices and pickled or salted fruits.
[c]Vegetables including fresh, frozen, cooked, canned, or dried vegetables and legumes, excluding starchy vegetables such as potatoes and corn, vegetable juices, and pickled or salted vegetables.
[d]Sugar-sweetened beverages were defined as beverages with ≥50 kcal per 8oz (237g), including carbonated beverages and fruit drinks, excluding 100% juices.
[e]Unprocessed red meats including beef, lamb, and goat, excluding poultry, fish, eggs, and all processed meats.
[f]Processed meats including meats preserved by smoking, curing, salting including sausages or luncheon meats.
[g]Intake of Eicosapentaenoic acid and Docosahexaenoic acid.
