# Supplementary material for: Cardiometabolic deaths attributable to poor diet among Kuwaiti adults
Source: PLoS One. 2022 Dec 15;17(12):e0279108. doi: 10.1371/journal.pone.0279108 (PMC9754186; doi:10.1371/journal.pone.0279108)
Supplement: S2 Table — (PDF) [file pone.0279108.s005.pdf]

S2 Table. Relative Risks[a] (RR) of the relationship between dietary factors and cardiometabolic outcomes

Dietary factor	Cardiometabolic outcome	Unit of RR	RR (95% CI) by age group			
			25-34 y	35-44 y	45-54 y	55+ y
**Fruits**, g/d	CHD	per 100 g/d	0.92 (0.87-0.97)	0.92 (0.87-0.97)	0.93 (0.89-0.97)	0.95 (0.91-0.98)
	Ischemic stroke		0.83 (0.76-0.90)	0.83 (0.77-0.90)	0.86 (0.80-0.92)	0.89 (0.83-0.94)
	Hemorrhagic stroke		0.63 (0.49-0.81)	0.64 (0.5-0.82)	0.69 (0.56-0.84)	0.75 (0.58-0.91)
**Vegetables**, g/d	CHD	per 100 g/d	0.93 (0.89-0.97)	0.93 (0.90-0.97)	0.94 (0.91-0.97)	0.96 (0.93-0.98)
	Ischemic stroke		0.76 (0.64-0.9)	0.77 (0.66-0.9)	0.80 (0.70-0.92)	0.84 (0.74-0.95)
	Hemorrhagic stroke		0.76 (0.61-0.95)	0.77 (0.62-0.95)	0.80 (0.67-0.96)	0.84 (0.70-0.98)
**Nuts/seeds**, g/d	CHD	per 4.05 g/d	0.89 (0.85-0.93)	0.89 (0.85-0.93)	0.91 (0.87-0.94)	0.93 (0.90-0.96)
	Diabetes		0.95 (0.92-0.98)	0.95 (0.93-0.98)	0.96 (0.94-0.98)	0.97 (0.95-0.99)
**Whole grains**, g/d	CHD	per 50 g/d	0.95 (0.91-0.99)	0.95 (0.92-0.99)	0.96 (0.93-0.99)	0.97 (0.94-0.99)
	Ischemic stroke		0.88 (0.80-0.96)	0.88 (0.81-0.96)	0.90 (0.83-0.97)	0.92 (0.86-0.98)
	Hemorrhagic stroke		0.88 (0.80-0.96)	0.88 (0.81-0.96)	0.90 (0.83-0.97)	0.92 (0.86-0.98)
	Diabetes		0.83 (0.76-0.90)	0.83 (0.77-0.90)	0.86 (0.80-0.92)	0.89 (0.83-0.94)
**Unprocessed red meats**, g/d	Diabetes	per 100 g/d	1.3 (1.05-1.60)	1.29 (1.05-1.57)	1.24 (1.04-1.47)	1.18 (1.04-1.31)
**Processed meats**, g/d	CHD	per 50 g/d	1.62 (1.17-2.18)	1.58 (1.16-2.11)	1.47 (1.14-1.88)	1.14 (1.14-1.54)
	Diabetes		1.86 (1.38-2.46)	1.81 (1.36-2.37)	1.65 (1.30-2.08)	1.46 (1.27-1.65)
**Sugar-sweetened beverages**[b], 8-oz servings/d	Increased BMI (BMI<25 kg/m^2 at baseline)[2]	per 8 oz/d	0.10 kg/m^2 (0.05 -0.15)	0.10 kg/m^2 (0.05 -0.15)	0.10 kg/m^2 (0.05 -0.15)	0.10 kg/m^2 (0.05 -0.15)
	Increased BMI (BMI ≥25 kg/m^2 at baseline)[2]		0.23 kg/m^2 (0.14-0.32)	0.23 kg/m^2 (0.14-0.32)	0.23 kg/m^2 (0.14-0.32)	0.23 kg/m^2 (0.14-0.32)
	BMI-mediated effect on CHD	per 5 kg/m^2 increa se in BMI	1.79 (1.56-2.06)	1.66 (1.51-1.84)	1.55 (1.46-1.64)	1.40 (1.38-1.43)
	BMI-mediated effect on HHD		2.30 (0.66-7.95)	2.15 (0.80-5.78)	2.02 (0.97-4.21)	1.59 (1.45-1.74)
	BMI-mediated effect on ischemic stroke		2.09 (1.81-2.40)	1.86 (1.67-2.08)	1.67 (1.53-1.81)	1.43 (1.37-1.49)
	BMI-mediated effect on hemorrhagic stroke		3.04 (2.24-4.11)	2.54 (1.96-3.28)	2.10 (1.66-2.66)	1.63 (1.45-1.81)
	BMI-mediated effect on diabetes		3.55 (2.41-5.23)	3.07 (2.28-4.15)	2.66 (2.15-3.30)	2.19 (2.10-2.28)
**Polyunsaturated fats replacing carbohydrates**, % energy	CHD	per 5% energy/d	0.86 (0.79-0.92)	0.86 (0.8-0.93)	0.88 (0.83-0.94)	0.91 (0.86-0.96)

**Polyunsaturated fats replacing saturated fats**, % energy	CHD	per 5% energy/d	0.87 (0.81-0.93)	0.87 (0.82-0.94)	0.89 (0.84-0.95)	0.92 (0.87-0.96)
**Seafood omega-3 fats**, mg/d	CHD	per 100 mg/d	0.79 (0.70-0.88)	0.80 (0.71-0.89)	0.82 (0.75-0.90)	0.86 (0.79-0.93)
**Sodium**[c], mg/d	Main effect on SBP among normotensive	per 2300 mg/d	1.64 mmHg (-0.19-3.46)	2.69 mmHg (1.15-4.23)	3.74 mmHg (2.30-5.17)	5.23 mmHg (3.57-6.89)
	Additional effect on SBP among hypertensive		1.87 mmHg (0.12-3.63)	1.87 mmHg (0.12-3.63)	1.87 mmHg (0.12-3.63)	1.87 mmHg (0.12-3.63)
	SBP-mediated effect on CHD	per 10 mmHg increase in SBP	1.81 (1.29-2.56)	1.68 (1.29-2.20)	1.56 (1.29-1.89)	1.40 (1.31-1.48)
	SBP-mediated effect on HHD		3.29 (3.00-3.60)	2.86 (2.67-3.06)	2.49 (2.37-2.61)	2.04 (2.01-2.07)
	SBP-mediated effect on RHD		1.28 (1.11-1.47)	1.24 (1.11-1.38)	1.20 (1.11-1.30)	1.15 (1.11-1.20)
	SBP-mediated effect on CMM		1.51 (1.39-1.64)	1.44 (1.35-1.54)	1.37 (1.31-1.43)	1.28 (1.25-1.31)
	SBP-mediated effect on AF		1.51 (1.39-1.64)	1.44 (1.35-1.54)	1.37 (1.31-1.43)	1.28 (1.25-1.31)
	SBP-mediated effect on AA		1.62 (1.45-1.80)	1.53 (1.40-1.66)	1.44 (1.36-1.53)	1.33 (1.30-1.36)
	SBP-mediated effect on PVD		1.51 (1.39-1.64)	1.44 (1.35-1.54)	1.37 (1.31-1.43)	1.28 (1.25-1.31)
	SBP-mediated effect on endocarditis		1.45 (1.26-1.67)	1.39 (1.24-1.55)	1.33 (1.22-1.44)	1.24 (1.20-1.29)
	SBP-mediated effect on other cardiovascular and circulatory		1.51 (1.39-1.64)	1.44 (1.35-1.54)	1.37 (1.31-1.43)	1.28 (1.25-1.31)
	SBP-mediated effect on Ischemic stroke		2.30 (2.07-2.56)	2.05 (1.89-2.22)	1.83 (1.72-1.93)	1.55 (1.51-1.59)
	SBP-mediated effect on hemorrhagic stroke		2.25 (1.67-3.04)	2.11 (1.50-2.98)	1.89 (1.43-2.51)	1.57 (1.44-1.71)

Abbreviations: AA, aortic aneurysm; AF, atrial fibrillation and flutter; BMI, body mass index; CHD, coronary heart disease; CI, confidence interval; CMM, cardiomyopathy and myocarditis; HHD, hypertensive heart disease; RHD, rheumatic heart disease; RR, relative risk; SBP, systolic blood pressure; PVD, peripheral vascular disease.

[a]RRs and 95% CIs of dietary factors on cardiometabolic outcomes from published meta-analyses of clinical trials and prospective studies [5, 6].

[b]The effect of sugar-sweetened beverages (SSBs) on cardiometabolic outcomes was estimated by combining the effect of SSBs on change in BMI (direct effect) and the effect of BMI on cardiometabolic outcomes (coronary heart disease, hypertensive heart disease, stroke, and diabetes) (indirect effect).

[c]The effect of sodium on cardiometabolic outcomes was estimated by combining the effect of sodium on change in SBP (direct effect) and the effect of SBP on cardiometabolic outcomes (coronary heart disease, hypertensive heart disease, other cardiovascular diseases, and stroke) (indirect effect).
