# Supplementary material for: Cardiometabolic deaths attributable to poor diet among Kuwaiti adults
Source: PLoS One. 2022 Dec 15;17(12):e0279108. doi: 10.1371/journal.pone.0279108 (PMC9754186; doi:10.1371/journal.pone.0279108)
Supplement: S3 Table — (PDF) [file pone.0279108.s006.pdf]

S3 Table. Cardiometabolic deaths[a] in Kuwait in 2009, total and by age and sex

	Total	25-34 y		35-44 y		45-54 y		55+ y	
**Cardiometabolic disease**	**25+ y**	**Men**	**Women**	**Men**	**Women**	**Men**	**Women**	**Men**	**Women**
Coronary heart disease	1169	39	9	126	12	277	26	470	210
Ischemic stroke	362	10	4	12	8	23	8	150	147
Hemorrhagic stroke	137	7	5	20	6	26	11	30	32
Other stroke	3	0	0	1	0	0	0	0	2
Total stroke	*502*	*17*	*9*	*33*	*14*	*49*	*19*	*180*	*181*
Hypertensive heart disease	92	0	2	0	1	5	3	31	50
Aortic aneurysm	4	0	0	0	0	0	1	2	1
Rheumatic heart disease	5	1	1	2	0	0	0	0	1
Endocarditis	0	0	0	0	0	0	0	0	0
Cardiomyopathy and myocarditis	19	0	2	0	1	1	1	10	4
Atrial fibrillation and flutter	5	0	0	0	0	0	0	1	4
Peripheral vascular disease	0	0	0	0	0	0	0	0	0
Other cardiovascular and circulatory diseases	25	3	0	1	1	3	0	8	9
Total cardiovascular disease	*1259*	*60*	*23*	*162*	*29*	*335*	*50*	*702*	*460*
Diabetes	201	2	1	2	4	8	9	89	86
Total cardiometabolic deaths	***2022***	***62***	***24***	***164***	***33***	***343***	***59***	***791***	***546***

[a]Disease-specific deaths in Kuwait in 2009 from the World Health Organization mortality database [7] and coded according to the official International Classification of Disease, Tenth Revision (ICD-10). Data obtained for mortality due to coronary heart disease (I20-I25); hypertensive heart disease (I11); aortic aneurysm (I71); rheumatic heart disease (I01, I020, I05-I09); endocarditis (I33); cardiomyopathy and myocarditis (I40, I42); atrial fibrillation and flutter (I48); peripheral vascular disease (I702, I73); other cardiovascular and circulatory diseases (I00, I1029, I27, I28, I30-I32, I34-I39, I47, I708, I72, I77-I80, I82-I84, I86, except I271, I312, I313); Ischemic stroke (I63, I65-I67, I693, G45); hemorrhagic stroke (I60-I62, I690-I692, I674); other stroke (I64, I694, I698); and, (3) diabetes (E10-E14, except E102, E112, E122, E132).
