# Supplementary material for: Cardiometabolic deaths attributable to poor diet among Kuwaiti adults
Source: PLoS One. 2022 Dec 15;17(12):e0279108. doi: 10.1371/journal.pone.0279108 (PMC9754186; doi:10.1371/journal.pone.0279108)
Supplement: S4 Table — (PDF) [file pone.0279108.s007.pdf]

S4 Table. Estimated cardiometabolic deaths (per 100,000 adults[a]) attributable[b] to suboptimal diet among Kuwaiti adults aged ≥25 years in 2009, total and by sex

**Outcome by dietary factor**	Total N (95% UI)	Men N (95% UI)	Women N (95% UI)	25-34 y N (95% UI)	35-44 y N (95% UI)	45-54 y N (95% UI)	55+ y N (95% UI)
**Overall diet**[c]							
CHD	184 (172-194)	327 (303-350)	70 (63-76)	29 (27-30)	97 (85-106)	287 (253-315)	553 (499-614)
Hypertensive heart disease	6 (4-7)	5 (3-6)	6 (4-8)	1 (0-1)	0 (0-0)	2 (2-4)	27 (21-35)
Ischemic stroke	46 (43-55)	57 (48-65)	38 (31-44)	8 (7-8)	13 (11-14)	26 (21-28)	207 (176-238)
Hemorrhagic stroke	24 (21-25)	32 (29-35)	16 (14-18)	8 (7-8)	20 (17-21)	36 (31-39)	53 (44-60)
Diabetes	21 (18-32)	25 (20-48)	18 (14-21)	2 (1-2)	4 (3-5)	11 (10-16)	96 (79-163)
Total CMD	*290 (273-330)*	*462 (427-543)*	*153 (142-163)*	*50 (46-54)*	*136 (124-159)*	*367 (328-451)*	*963 (881-1239)*
**Fruits**, <300 g/d							
CHD	26 (18-34)	48 (31-66)	9 (5-14)	6 (3-9)	20 (9-31)	42 (18-70)	66 (34-98)
Stroke	29 (25-34)	39 (31-46)	22 (17-28)	9 (7-10)	21 (16-24)	32 (22-39)	91 (69-115)
Total CMD	*56 (46-65)*	*86 (69-105)*	*31 (24-39)*	*15 (12-18)*	*41 (28-52)*	*74 (49-102)*	*155 (117-194)*
**Vegetables**, <400 g/d							
CHD	22 (16-28)	39 (27-54)	8 (4-12)	5 (3-8)	15 (7-22)	34 (17-53)	58 (31-91)
Stroke	31 (23-38)	40 (27-53)	24 (15-33)	9 (6-10)	16 (9-21)	26 (10-36)	113 (69-155)
Total CMD	*52 (43-63)*	*78 (61-98)*	*31 (22-41)*	*13 (10-16)*	*30 (21-40)*	*60 (39-81)*	*174 (123-224)*
**Nuts/seeds**, <20.2 g/d							
CHD	79 (65-91)	139 (114-167)	30 (22-38)	13 (10-16)	42 (28-56)	124 (84-161)	241 (179-299)
Diabetes	6 (4-8)	7 (4-10)	5 (3-8)	1 (0-1)	1 (0-1)	4 (2-5)	28 (19-39)
Total CMD	*84 (71-97)*	*146 (120-174)*	*35 (27-43)*	*13 (10-16)*	*43 (28-57)*	*128 (86-165)*	*269 (208-329)*
**Whole grains**, <125 g/d							
CHD	18 (11-25)	32 (18-49)	6 (3-9)	3 (1-6)	11 (3-19)	30 (7-52)	48 (18-78)
Stroke	18 (13-22)	24 (16-31)	13 (8-18)	5 (3-6)	9 (6-12)	16 (11-22)	63 (38-87)
Diabetes	9 (7-11)	11 (7-14)	8 (5-11)	1 (1-1)	2 (1-2)	5 (4-7)	43 (30-56)
Total CMD	*45 (36-54)*	*66 (50-84)*	*27 (21-34)*	*9 (6-12)*	*22 (14-31)*	*52 (28-75)*	*154 (114-196)*
**Sugar-sweetened beverages**, >0 8-oz servings/d							
CHD	38 (30-46)	72 (56-92)	10 (7-13)	13 (9-17)	30 (18-45)	65 (39-99)	79 (51-113)
Stroke	1 (1-2)	2 (2-3)	1 (1-1)	1 (1-1)	1 (1-2)	1 (1-2)	3 (3-4)
Diabetes	5 (4-7)	7 (4-11)	4 (2-6)	1 (0-1)	2 (1-3)	4 (2-6)	22 (13-36)
Total CMD	*45 (37-54)*	*81 (65-103)*	*15 (12-19)*	*15 (11-20)*	*33 (22-47)*	*71 (44-103)*	*106 (75-142)*
**Unprocessed red meat**, >14.3 g/d							
Diabetes	3 (2-5)	4 (1-8)	3 (1-4)	0 (0-1)	0 (0-1)	1 (0-2)	16 (6-28)
Total CMD	*3 (2-5)*	*4 (1-8)*	*3 (1-4)*	*0 (0-1)*	*0 (0-1)*	*1 (0-2)*	*16 (6-28)*
**Processed red meat**, >0 g/d							
CHD	11 (3-116)	20 (4-257)	4 (1-14)	8 (1-33)	11 (2-110)	2 (0-341)	5 (0-609)
Diabetes	2 (1-22)	2 (1-46)	2 (1-5)	1 (0-2)	3 (0-5)	1 (0-11)	1 (0-115)
Total CMD	*16 (4-125)*	*28 (6-275)*	*6 (1-16)*	*10 (2-33)*	*14 (3-113)*	*4 (1-342)*	*9 (1-611)*
**Sodium**, >2000 mg/d							
CHD	32 (24-41)	54 (39-72)	14 (10-20)	3 (1-7)	14 (6-27)	52 (28-83)	102 (74-140)

Stroke	18 (16-22)	22 (17-27)	16 (12-20)	3 (2-4)	7 (5-10)	16 (12-21)	74 (58-92)
Total CMD	*57 (49-66)*	*81 (65-100)*	*37 (31-44)*	*7 (5-11)*	*22 (13-35)*	*71 (48-103)*	*208 (174-250)*
**PUFA replacing carbohydrates**, <11% energy/d							
CHD	36 (28-44)	64 (47-81)	13 (8-18)	6 (3-8)	21 (11-28)	58 (33-83)	106 (67-144)
Total CMD	*36 (28-44)*	*64 (47-81)*	*13 (8-18)*	*6 (3-8)*	*21 (11-28)*	*58 (33-83)*	*106 (67-144)*
**Seafood omega-3 fats**, <250 mg/d							
CHD	43 (23-64)	73 (32-121)	18 (9-28)	8 (2-14)	21 (3-55)	84 (10-161)	105 (47-212)
Total CMD	*43 (23-64)*	*73 (32-121)*	*18 (9-28)*	*8 (2-14)*	*21 (3-55)*	*84 (10-161)*	*105 (47-212)*

Abbreviations: CHD, coronary heart disease; CMD, cardiometabolic diseases; PUFA, polyunsaturated fatty acids; UI, uncertainty intervals

[a]Per 100,000 population.

[b]Attributable deaths were calculated by multiplying the total number of disease-specific deaths in Kuwait in 2009 with the stratum-specific population attributable fraction.

[c]For the overall diet, the population attributable fraction was estimated based on the joint (multiplicative) population attributable fraction for ten dietary factors: fruits, vegetables, whole grains, nuts/seeds, seafood omega-3 fats, PUFA as a replacement of saturated fats or carbohydrates, sodium, sugar-sweetened beverages, processed meat, and unprocessed red meat.
